# Apoptosis and cell cycle arrest of bone marrow cells by green-synthesized silver but not albumin nanoparticles

**DOI:** 10.1016/j.toxrep.2025.101960

**Published:** 2025-02-13

**Authors:** Ehdaa Eldabousy, Lotfy Habbak, Ayman Hyder

**Affiliations:** Faculty of Science, Damietta University, New Damietta 34517, Egypt

**Keywords:** Green silver nanoparticles, Albumin nanoparticles, Bone marrow cells, Apoptosis, Cell cycle

## Abstract

Metallic nanoparticles (NPs) made by traditional means have a deleterious effect on bone marrow (BM) cells. Alternatively, green-synthesized NPs are cost-effective, ecofriendly, and may be less toxic. Also, albumin is a biocompatible blood protein involved in several physiological processes, employed in drug delivery without posing adverse effects, and is thought to be ideal NPs or coating for reducing the metallic NP’s toxicity. We prepared albumin NPs (AlbNPs), biosynthesized silver NPs (AgNPs) using the metabolite of the *Escherichia coli* D8 strain and coated them with albumin (Ag/AlbNPs). These NPs were characterized and intraperitoneally administered to rats to compare their effect on rat BM cells. The flow cytometry results revealed that AgNPs significantly reduced viability, increased apoptosis, downregulated the antiapoptotic *Bcl2* gene expression, and upregulated the apoptotic genes *Bax* and *p53* in BM cells, while treatment with AlbNPs maintained these parameters. Principally, AgNPs caused significant DNA fragmentation, since all parameters observed by the comet assay (tail length, tail DNA content, tail moment, and olive moment) were significantly higher in AgNP-treated groups than in control and AlbNP-treated groups. Investigation of the cell cycle revealed that treatment with AgNP, but not AlbNPs, downregulated the expression of the regulatory genes *Cdk2, Cdk4*, and the cyclins A1 (*Ccna1*) and D1 (*Ccnd1*), which resulted in the arrest of the progression of the cell cycle at GO/G1, as demonstrated by flow cytometry. Coating AgNPs with albumin increased their size, and decreased their intracellular concentration, resulting in reduced apoptosis and cell cycle arrest. However, these results for the Ag/AlbNP-treated group were still not comparable to those treated with pure AlbNPs. In conclusion, in contrast to AlbNPs, green AgNPs are toxic to bone marrow cells. Their coating with albumin, however, reduces this toxicity. To avoid this metal NP toxicity, it is recommended to use compatible degradable NPs instead of metal NPs for medication delivery to BM.

## Highlights

• The toxicity of metal (silver) and polymer (albumin) nanoparticles was compared in bone marrow cells.
• AgNPs, but not albumin NPs, increased apoptosis and apoptotic *Bax* and *p53* expressions*.*
• AgNPs, but not albumin NPs, arrested the progression of the cell cycle at GO/G1.
• Coating AgNPs with albumin increased their size, resulting in reduced apoptosis and cell cycle arrest.
• Green AgNPs are toxic to bone marrow cells.

## Graphical Abstract

**Figure:**
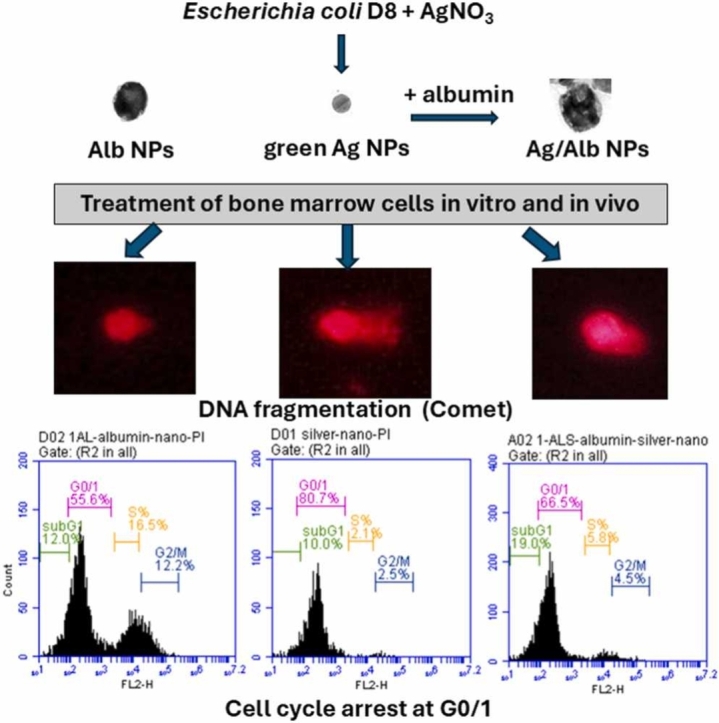

## 1 Introduction

Nanotechnology is one of the most significant and innovative sciences in this era. The goal of this science is to produce nanoparticles and use them in a variety of applications including medicine [1], genetic engineering [2], water purification [3], and bio-detection of pathogens [4]. Nanoparticles have been classified into inorganic metallic NPs, which are metals such as cobalt, gold, lead, zinc, silver, or their oxides, non-metallic carbon-based NPs such as graphene and dendromer NPs, polymeric NPs such as chitosan, lipid and albumin NPs, and others like silica and hydroxyapatite NPs [5], [6].

NPs’ toxicity against bone marrow (BM) cells is an important concern. In fact, even when bone marrow is not the primary target for NP-treatments, it can still be affected [7]. Once administered, NPs can circulate throughout the body, including reaching the BM. This can lead to interactions with BM cells. NP-exhibited toxicity against bone marrow cells potentially leads to issues with blood cell production [8]. Also, NPs might induce inflammation in various tissues, including bone marrow [9]. Chronic inflammation can negatively affect the bone marrow environment and its ability to function properly. Certain NPs have been shown to cause genetic damage [10]. This could potentially affect the DNA of bone marrow cells, leading to long-term consequences.

The toxicity of nanoparticles against BM cells is a multi-faceted issue influenced by several factors. Different NP materials exhibit varied toxicological profiles. For example, metal-based NPs may induce cytotoxicity through oxidative stress, while polymeric NPs might have different pathways [11]. Higher concentrations, smaller sizes of NPs, and prolonged exposure typically correlate with increased toxicity. Smaller size of NPs increases the surface area-to-volume ratio, enhancing the exposure of BM cells to the reactive sites on the NPs, which can lead to higher toxicity [12]. Surface Modifications as coatings can alter the NP's interaction with cells [13]. The biological environment, including pH, can functionally alter many cell systems [14]. It influences NP stability, aggregation, and surface interactions, thereby affecting toxicity [15].

Of interest, silver NPs are metallic nanoparticles present in daily used products including medical and health care products [16], cosmetics [17], and food packaging [18]. Despite the rapid invasion of AgNPs in all essential industries they still pose a threat to human health and the environment. This danger lies in the toxicity of these AgNPs [19]. Previous studies on humans and animals confirmed the toxicity of AgNPs in liver [20], intestine [21], lung [22], heart [23], and reproductive organs [24].

The detrimental toxic effects of AgNPs on rat bone marrow hematopoietic cells were verified by *in vitro* studies. These effects were reported to be initiated by reducing cell viability [25], overproduction of reactive oxygen species (ROS) [26], DNA damage [27], cell cycle arrest [28], and apoptosis [19]. It is known that the conventional physical and chemical methods used to generate nanoparticles in the past required time, effort, energy, money, and resources [29]. Additionally, these methods generate extremely hazardous nanoparticles that negatively impact the environment [17]. Many efforts are being made towards the green biosynthesis of nanoparticles that requires no specific chemical, or radiation hazards and creates less toxic, distinctive affordable, and eco-friendly nanoparticles prepared by biological sources like bacteria [30], fungi [31], algae [32], and plant species [33].

On the other hand, nonmetallic or organic nanoparticles like albumin nanoparticles have a variety of applications in scientific research and medical field [34]. Since albumin is a non-toxic versatile protein in the body and has a high portability for binding drugs [35], numerous studies have shown the effectiveness of albumin nanoparticles (AlbNPs) as drug carriers [36], [37]. The cytotoxic assay using different cells and AlbNPs supports the nontoxic action of AlbNPs to mammalian cells [38], [39]. In vivo, treatment with AlbNPs was proven safe for the liver [40] and kidney [41]. However, only one study reported that AlbNPs induce skin histopathological changes and inflammation in mice [42]. However, little is known about the effect of albumin NPs on bone marrow cells. As well, a comparative study of cellular effects between metal and organic NPs, especially in bone marrow cells, has not yet been undertaken.

Information about the response of bone marrow cells to different kinds of nanoparticles is scarce. In the present study, it is assumed that organic biodegradable nanoparticles may have less cytotoxicity on bone marrow cells than metal nanoparticles, even if those were greenly synthesized. Here we have recruited *Escherichia coli* D8 whose crude metabolites act as strong bio-reductant agents to biosynthesize green silver nanoparticles. The *in vitro* uptake and toxicity of these green-synthesized AgNPs in bone marrow cells have been investigated. These AgNPs have been coated with albumin to investigate whether this coating would reduce their toxicity, if any. The effect of both above-mentioned groups has been compared with that of pure albumin NPs on bone marrow cells *in vitro* and *in vivo*.

## 2 Material and methods

### 2.1 Sub-culturing of Escherichia coli D8 and production of metabolites

*Escherichia coli* D8 strain, isolated from a sewage water stream located at Damietta, Egypt, is kindly provided by Dr. M. El-Zahed, Department of Microbiology, Faculty of Science, Damietta University, who classically identified the strain and confirmed this identification by the 16S rRNA sequence analysis. This sequence is deposited in the GenBank under accession number MF062579.1. The *Escherichia coli* D8 strain utilized in the biosynthesis of AgNPs was cultured on nutrient agar medium for activation and incubated at 37 °C for 24 hours in a static incubator. The growing colonies of bacteria were picked up and grew in nutrient broth (NB) medium and incubated at 37°C for 48 hours at 150 rpm. The bacterial crude metabolite (Supplementary Fig. S1) was collected by centrifuging at 5000 rpm for 20 min [24].

### 2.2 Green synthesis of silver nanoparticles

The method reported by Elsharawy et al. [24] was followed for the biosynthesis of AgNPs using the metabolite of different bacteria. Briefly, 6.5 mL of 10 mM silver nitrate and 3.5 mL of *E. coli* metabolite were combined in a conical flask. To trigger the reaction, the solution was kept in sunlight for 10 minutes. As a sign that AgNPs are being biosynthesized, the reaction's color turned brown (Supplementary Fig. S1). Agents in the bacterial metabolite can reduce silver ions to brown-colored silver metal. The solution containing the produced silver nanoparticles was kept at room temperature in suspension form. As a control, a sample is kept in darkness.

### 2.3 Preparation of albumin nanoparticles

Albumin nanoparticles were prepared according to the method of Bronze-Uhle et al. [43] with modifications. Briefly, two hundred mg of bovine serum albumin (lyophilized powder, 98 %, Merck) were dissolved in 2 mL of deionized water using a magnetic stirrer set at 600 rpm for 20 minutes at 25 °C. After dissolving, 5 mL of ethanol (1 mL/min) and 0.16 mL of 8 % (v/v) glutaraldehyde were added to cross-link the dissolved albumin. The mixture was then agitated at 25°C for 20 hours before being centrifuged three times for 25 min at 13,000 rpm to get rid of dissolved and all other chemicals. The NPs were redispersed in 10 mL of deionized water using an ultrasonication bath for 10 minutes following each centrifugation cycle. The produced sample was kept as a suspension at 4 °C.

### 2.4 Preparation of albumin coated silver nanoparticles

One gram of bovine serum albumin was dissolved in 10 mL deionized water as mentioned above until the albumin was completely dissolved. Following dissolution AgNPs were added to the solution in a ratio of 7:1 (AgNPs:BSA) [44]. An electric mixer was used to combine this solution for 18 hours at room temperature and then store it as a suspension at room temperature.

### 2.5 Characterization of the prepared nanoparticles

#### 2.5.1 Transmission electron microscope analysis

On a copper-coated grid a little drop of the produced nanoparticles' aqueous solution was applied. It was subsequently impregnated with lead citrate and uranyl acetate for staining and the shape and diameter of AgNPs, AlbNPs, and Ag/AlbNPs were imaged using transmission electron microscope (TEM, JEOL JEM-2100, Japan).

#### 2.5.2 Ultraviolet visible (UV-Vis) spectrophotometric analysis

Green biosynthesized NPs were observed for their UV–Vis absorption spectra. A double beam UV–vis spectrophotometer was used to measure the solutions after they had been diluted 1:10. The bacterial metabolite was boiled to create a controlled sample which was then placed in the dark to prevent the reducing agent from acting. Distilled water was used as a blank.

#### 2.5.3 Fourier transforms infrared spectroscopy (FT-IR) study

The absorbance of the greenly synthesized nanoparticles of Fourier infrared radiation was observed using an FT-IR-4100 type A in the diffuse reflectance mode which scanned the 400–4000 cm^−1^ spectrum at a resolution of 4 cm^−1^. The apparatus recorded the stretching peaks and vibrations of the major functional groups found in the produced biomolecules.

#### 2.5.4 Zeta potential analysis

The produced NPs’ colloidal solution was subjected to stability and surface charge detection using a Zeta Potential Analyzer (Malvern Zeta-sizer, Nano-zs90, Japan). The technique relies on the size and concentration of the colloidal solution. Therefore, before measuring, and to prevent a cloudy suspension of the produced nanoparticles, samples were diluted according to particle size to 1:10 (<100 nm) or 1:100 (>100 nm) with distilled water [45].

### 2.6 Animal experiments

Animal experiments comply with the ARRIVE guidelines and were carried out in accordance with the U.K. Animals (Scientific Procedures) Act, 1986 and associated guidelines, and EU Directive 2010/63/EU for animal experiments. The Damietta University institutional ethical committee for animal research authorized and approved all the procedures and animal handling (Approval number and date: DuREC No 67 on Feb 27, 2023). Twenty-five male Wistar rats with an average weight of 120 −180 g were used in this study. They were housed with food and drink *ad libitum* in well-ventilated cages with standard climatic parameters of 45–55 % humidity, 12 hours of darkness and light, and a temperature of 25 ± 2°C. Animals were divided into 4 groups of five animals each: a control group received no NPs, an AgNPs group, an AlbNPs group, and an Ag/AlbNPs-treated group. For three weeks, the animals received intraperitoneal injections of 100 mg/kg NPs [24]. The injections were given three times a week, day after day. The last 5 rats were used in *in vitro* experiments. Rats were euthanized with intraperitoneal injection of ketamine (50 mg/kg) / xylazine (5 mg/kg) solution. From each group, bone marrow cells were extracted from the 4 long bones (2 femurs and 2 tibias).

For *In vitro* experiments, bone marrow cells were isolated from the last 5 animals. The 4 long bones (2 femurs and 2 tibias) were excised, bone heads were removed, and bone marrow flushed using PBS. After washing and centrifugation, cells were incubated for 2 hours at 37 °C with DMEM medium containing 10 % FCS. The culture medium contained either AgNPs (2 µg/mL) [46], AlbNPs (1 mg/mL) [47] and Ag/AlbNPs (1 mg/mL) [48]. The control group was cultured in the same medium without adding any NP preparation.

### 2.7 Intracellular nanoparticle observation

The cellular uptake of NPs was examined qualitatively ultrastructural observation and quantitatively by the intracellular determination of silver metal concentration. For ultrastructural analysis, cell pellets were fixed for 20 min in 2.5 % glutaraldehyde then washed with 0.08 M phosphate buffer at 20 °C and centrifuged. After fixation, cell pellets were impregnated in osmium tetroxide for 15 min. Dehydration was done by ethanol then samples were embedded in Spurr’s resin. An ultra-microtome was used to create the ultra-sections, which were then stained with lead citrate and uranyl acetate and inspected under a transmission electron microscope (JEOL JEM-2100, Japan).

For quantitative analysis of intracellular silver content, cell pellets were dried in an oven at 60 °C. The cell pellet was digested using both cold and hot digestion. Cold digestion was performed for one hour at room temperature using a mixture of nitric acid (1 M) and perchloric acid (1 M) with a ratio of 2:1 v/v. Hot digestion was performed using a hot plate at 120 °C. The sample was allowed to cool before being diluted with distilled water to a volume of 25 mL. The silver concentration was then determined using the PerkinElmer PinAAcle 500 flame atomic absorption spectrometer.

### 2.8 Flow cytometric analysis of cell cycle

Following cell isolation and PBS washing the cell pellet was fixed in 70 % ethanol and incubated at 4 °C for the whole night. The cell pellet was 2x washed in PBS and spun at 850 g in a centrifuge then resuspended in 200 µL of staining solution (BD Biosciences, UK) (9.45 mL DPBS+500 µL 20X propidium iodide (PI)+ 50 µL 200X RNase). Both the forward scatter (FS) and side scatter (SS) were measured to identify single cells. Samples were analyzed by using an Accuri C6 flow cytometer (Becton Dickinson). Data in percentages of PI fluorescence was gathered using the Accuri C6 software.

### 2.9 Annexin V flow cytometric analysis of apoptosis

Using the Annexin V apoptosis kit (cat no. ab14085, Abcam, UK), cell pellets were suspended in 250 µL of binding buffer and 5 μL of fluorochrome-conjugated Annexin V was added to 100 μL of the cell suspension. This mixture was incubated for 15 minutes at room temperature in the dark, centrifuged, rinsed with 2 mL of 1x binding buffer. 5 μL of Propidium Iodide Staining Solution was incorporated into the cell suspension and this preparation was left at room temperature in the dark for 5 minutes. Samples were analyzed by using an Accuri C6 flow cytometer device (Becton Dickinson). The fluorescence data in percentages was collected using Accuri C6 software.

### 2.10 Cell viability test

Cells were incubated *in vitro* for 2 hours with different treatments. Directly after the incubation, they were centrifuged and suspended with 10 µL PBS before adding. 10 µL of trypan blue dye (0.4 %) (cat no. #72–57–1; Sigma). They were then incubated at room temperature for one minute before being examined under a light microscope.

### 2.11 Differential count test

Bone marrow is a heterogeneous tissue containing many cell types. It is assumed that not all these cells are affected similarly by NP toxicity. The flowcytometry estimation of apoptotic or necrotic cells could not determine which cell type is the most affected by different treatments. Bone marrow smears were prepared from different groups. These smears were fixed by covering them with absolute methanol for 30 seconds. Slides were then immersed in Giemsa stain for 30 seconds. Different types of hematopoietic stem cells were examined using a light microscope.

### 2.12 Comet assay

DNA damage was detected using the alkaline comet assay technique [49]. A known volume of cell suspension was mixed with 70 µL of 1 % low -melting point agarose at 37 °C. The mixture was transferred to microscopic slides that had been precoated with melted 1 % agarose. slides were dipped into a lysis solution consisting of 1 % Triton X-100 10 % dimethyl sulfoxide and 89 % lysis buffer that contained 10 mM Tris 2.5 mM NaCl and 100 mM Na_2_EDTA at a pH of 10. Following lysis, slides were put into a horizontal electrophoresis chamber supplemented with an alkaline mixture (1 mM 300 mM of Na_2_EDTA and NaOH respectively at pH > 14). The slides were kept in the dark for 15 minutes before being electrophoresed (1 V/cm, 20 min), neutralized in PBS for 5 minutes, dehydrated in methanol for 5 minutes, and scored using Gel Red stain. The percentage of DNA in the tail was assessed using Komet 6 software in 100 random nuclei (50 per replicate agarose gel) per sample.

### 2.13 Quantitative RT-PCR analysis

The expression of some genes involved in inflammation, apoptosis and cell cycle has been investigated by RT-PCR. Bone marrow cell RNA was extracted, and reverse transcribed to first strand cDNA using kits from Applied Biotechnology, Ismailia, Egypt. Quantitative RT-PCR analysis was made using SYBR Green (Applied Biotechnology, Ismailia, Egypt). The designed primer pairs and gene names and their GenBank accession numbers are listed in Table 1. Primers were designed using Primer3Plus software. Forward and reverse primers were designed to have approximately the same Tm, and exons-spanning primers were chosen. All expression data were normalized by relation to the corresponding expression of beta actin gene (*ACTB*), which was used as a housekeeping gene. Relative gene expression was calculated by 2^-ΔΔCt^ method [50].

**Table 1:** Primer sequences used for Quantitative RT-PCR.

Genes	Accession no	Forward primer sequence	Reverse primer sequence
Beta actin *(ACTB)*	NM_031144.3	AGCCTTCCTTCCTGGGTATG	TAGGAGCCAGGGCAGTAATC
*Cdk2*	NM_199501.2	CTCACTGGCATTCCTCTTCC	GCTGGAAGTCGGGTTAAGTG
*Cdk4*	NM_053593.3	GTGTACAAAGCCCGAGATCC	CTGGTCAGCCTCAGAGTTCC
Cyclin A1 *(Ccna1)*	NM_001011949.1	CAGGGAGAGAGGGCATAGTG	TCTCAGTCCTGATGCACACC
Cyclin D1 *(Ccnd1)*	NM_171992.5	CCCAACAACTTCCTCTCCTG	CCACTCCTGGGATAAAGCAC
*Bax*	NM_017059.2	ACCAAGAAGCTGAGCGAGTG	AGATGGTGAGTGAGGCAGTG
*Bcl2*	NM_016993.2	AGCTGTCACAGAGGGGCTAC	CTGAGCAGCGTCTTCAGAGA
*p53*	NM_030989.3	AGCACAGGAACCTGGAACTG	CTTCGGGTAGCTGGAGTGAG

### 2.14 Statistical analysis

Data are represented as mean ± SEM of number of samples N = 5. ANOVA was used for data analysis followed by student's *t*-test as a post hoc test, whenever ANOVA was significant. A p < 0.05 was considered statistically significant in all cases.

## 3 Results

### 3.1 Characterization of the biosynthesized nanoparticles

**Fig. 1:**
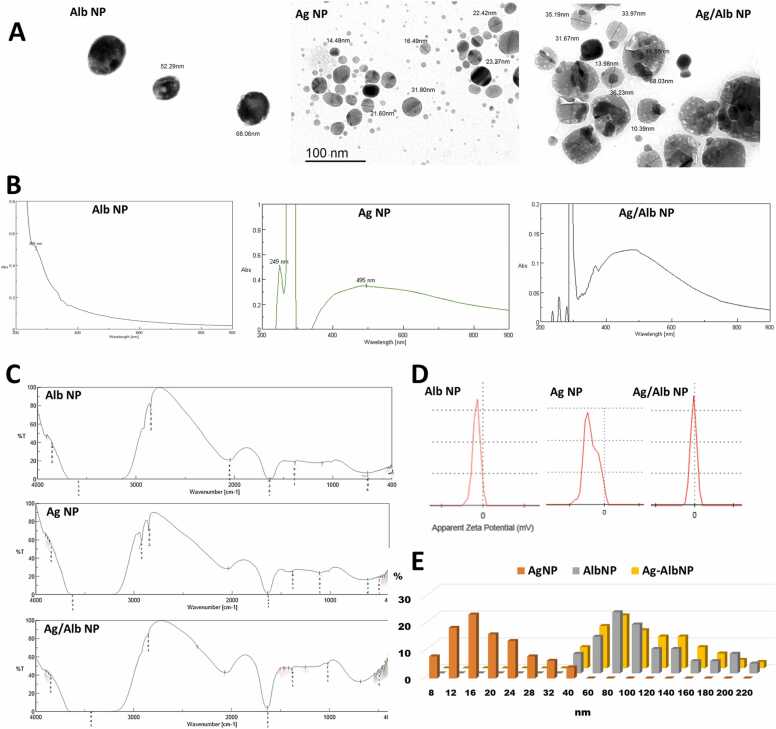
Characterization of differential nanoparticles: albumin nanoparticles (AlbNP) biosynthesized silver nanoparticles (AgNP) and albumin-coated silver nanoparticles (Ag/AlbNP). (A) The transmission electron micrographs compare the structures of different NPs. (B) The UV-Vis spectra of different NPs show absorption bands at 255 482 for AlbNPs and AgNPs respectively. Ag/AlbNPs showed a sharp peak at 249 nm and an absorption band at 495 nm. (C) FTIR analysis of different NPs. (D) Zeta potential distribution showing sharp peaks at −1.74 mv −19.6 mv and −9.21 mv for AlbNPs, AgNPs and Ag/AlbNP, respectively. (E) Size (nm) and frequencies (%) histogram of different NPs.

TEM micrographs revealed distinct structures for AlbNPs, AgNPs and Ag/AlbNPs (Fig. 1A). The generated AlbNPs had an irregular size distribution and an oval morphology. AgNPs had small sizes and mostly spheroid form. Ag/AlbNPs had an asymmetric hexagonal form and were larger in size.

The absorbance bands for the generated nanoparticles were displayed in (Fig. 1B). AlbNPs UV-Vis spectra revealed an absorption band at 255 nm. AgNPs exhibited an absorption band at 482 nm, whereas Ag/AlbNPs displayed an absorption band at 495 nm and a strong peak at 249 nm.

The FT-IR analysis (Fig. 1C) was performed to reveal the existence of functional groups that are distinct to the greenly biosynthetic process and stabilization of AgNPs. AgNPs' FT-IR analysis revealed bands at 3851 cm^−1^ and 3387 cm^−1^, which were attributed to the -OH and N-H stretch, which stands for protein, phenols, or alcohol. The >CH2 and -OCH3 C-stretch of an alkane or aldehyde was revealed by the absorption bands at 2919 and 2850 cm^−1^.The absorption band at 1632 cm-^1^ displays the amine's -NH bent. The nitrocompound's N-O stretch is represented by the peak at 1384 cm-1. The aliphatic amine's C-N stretch is indicated by the band seen at 1109 cm^−1^. The FT-IR spectra of AlbNPs and Ag/AlbNPs, respectively, showed a band at 3317 and 3464 cm^−1^, which were assigned to the stretching vibration of -OH and N-H of primary amine. The bands at 2851 cm^−1^ correspond to the HC=O stretching vibration of aldehyde. The bands at 1638 and 1635 cm^−1^ assigned to having an approximate vibration of (NH) C=O of amide. Bands between 1109 and 1017 cm^−1^ corresponding to C-O stretching of amino acid. Our research revealed that the interaction between silver and albumin and the creation of Ag/AlbNPs were validated by the presence of the same functional groups in AgNPs and AlbNPs samples.

Zeta potential for the generated nanoparticles was displayed in Fig. 1D. A sharp peak was seen for AlbNPs at −1.74 mv, for AgNPs at −19.6 mv, and for Ag/AlbNPs at −9.21 mv. The negative charge on their surfaces indicated a strong repulsion between the particles and stability of the solution.

Fig. 1E illustrates sizes and frequencies of the produced nanoparticles. The generated AlbNPs and Ag/AlbNPs ranged in size from 40 to 220 nm with an average of 130 ± 60.53 nm. The average size of AgNPs ranged from 8 to 40 nm with an average of 22.5 ± 10.67 nm (mean±SD).

### 3.2 Silver nanoparticles accumulate within bone marrow cells

**Fig. 2:**
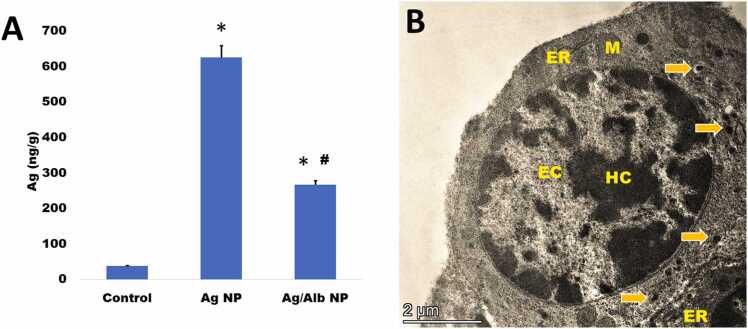
Intracellular detection of nanoparticles. (A) Quantitative analysis of silver content in bone marrow cells after *in vitro* incubation for 2 h with AgNPs and Ag/AlbNPs. The Ag concentration in the culture medium was 2 μg/mL, while albumin concentration was 1 mg/mL. The data is presented as means ± SEM. The “* ” denotes a significantly higher value than that of the control group and the “#” denotes a significantly lower value than that of the AgNPs group. (B) Transmission electron microscope of a lymphocyte of rat bone marrow treated *in vitro* with AgNPs (2 µg/mL) showed engulfed nanoparticles in lysosomes (arrows). ER = endoplasmic reticulum, M = mitochondria, EC =euchromatin, and HC =heterochromatin.

For this purpose, isolated hematopoietic bone marrow cells were briefly (2 hours) cultured *in vitro* in DMEM medium containing either AgNPs or albumin-coated AgNPs (Fig. 2A). An analysis of the intracellular silver concentration in bone marrow cells showed a significant difference between the groups (ANOVA p = 0.007). Both coated and uncoated AgNPs accumulated significantly in BM cells, when compared with the Ag concentration in untreated cells. Coating AgNPs with albumin decreased the penetration into BM cells, since Ag concentration was significantly lower in cells treated with Alb/AgNPs than in cells treated with uncoated AgNPs.

Silver NPs were also visualized in bone marrow cells processed for ultrastructural analysis. Fig. 2B shows a representative BM lymphocyte with a large nucleus, tiny euchromatic patches in contact with the nuclear envelope and heterochromatin clumps mostly located in the nuclear periphery. Deposited AgNPs engulfed by lysosomes were visible in the cytoplasm. Other cytopathological observations included also dilated cisternae of the endoplasmic reticulum. Taken together, the results show that AgNPs quickly accumulate in BM cells causing pathological changes in these cells.

### 3.3 Comparative effects of in vivo treatment with AgNPs vs. AlbNPs on BM cells

Animals were treated with AgNPs, AlbNPs, or albumin-coated AgNPs as described above. Bone marrow cells were isolated from the long bones. The effect of different NP-treatments on the BM cells was evaluated through the analysis of apoptosis, cell cycle, and differential count of rat bone marrow cells.

#### 3.3.1 Less apoptosis and genotoxicity in albumin NP- than in silver NP-treated BM cells

**Fig. 3:**
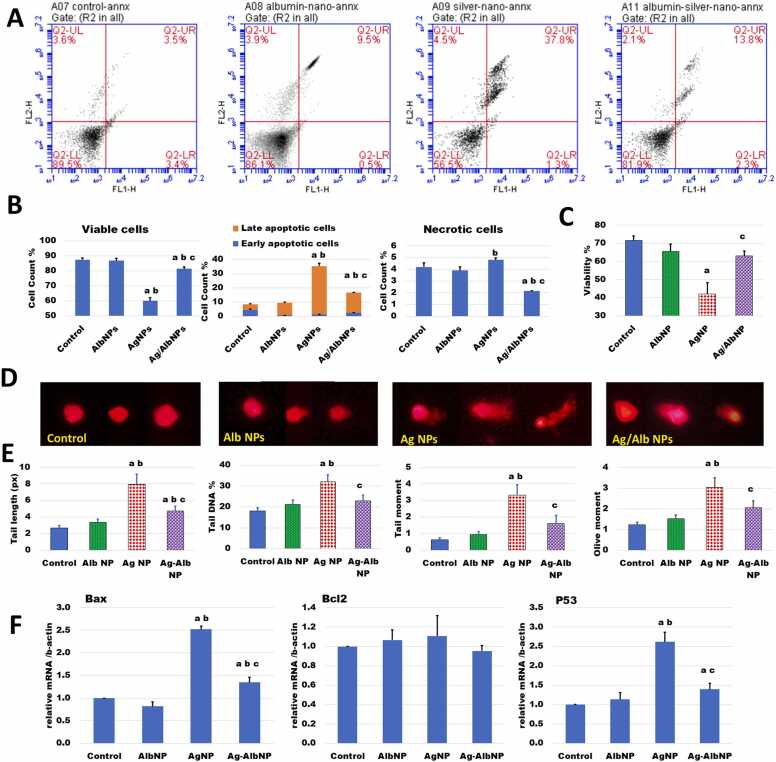
AgNPs but not AlbNPs induce apoptosis of bone marrow cells. Bone marrow was excised from rats ip. administered different NP preparations 3 times a week for 3 weeks, (A) Flow cytometry of Annexin-V/PI-stained cells was used to determine apoptosis induced by treatment of bone marrow cells with different NPs. LL: viable cells LR: early apoptosis UR: late apoptosis UL: necrosis. (B) Quantification of viable apoptotic and necrotic bone marrow cells after treatment with different NPs. The presented data is a summary of the flowcytometric analysis results. (C) Cell viability as determined by trypan blue vital staining of isolated bone marrow cells from untreated rats after a 2 h *in vitro* incubation with different NPs. (D) Photomicrographs of comet assay to assess *in vivo* DNA damage induced by NPs in bone marrow cells. (E) The quantitative analysis of comet assay parameters consists of the analysis of the comet tail length tail DNA tail moment and olive moment of DNA. (F) Expression of the apoptosis-related genes *p53, Bax*, and *Bcl2* as determined by RT-PCR. *Statistical analysis*: Data is presented as mean ± SEM of N = 5. ANOVA p < 0.05. The letters a, b, and c denote significantly different values from that of control AlbNPs and AgNPs groups, respectively (post hoc *t*-test).

To explore the role of the NPs under study in triggering apoptosis in BM cells, isolated cells were labeled with PI and Annexin V-FITC and subjected to apoptosis assay using flow cytometry (Fig. 3A and B). Apoptotic DNA damage was also measured by comet assay (Fig. 3D and E). Fig. 3A shows representative plots of flow cytometrical apoptosis analysis of BM cells from different nano-treatments. The percentage of viable, apoptotic, and necrotic cells of the investigated groups are presented in Fig. 3B.

The demonstrated effect on viability was similar to that observed in vitro (Fig. 3C), where intracellular accumulation of AgNPs reduced BM cell viability. After the 2 h-short-term *in vitro* culture, cell viability was directly examined by trypan blue vital stain. The cell viability findings showed a significant difference between the groups under study (ANOVA p = 0.001). The intergroup differences revealed that incubation with albumin NPs did not affect the cell viability, while incubation with AgNPs significantly decreased the viability. However, coating AgNPs with albumin restored this cell viability (Fig. 3C).

Like the *in vitro* results shown in Fig. 3C, the *in vivo* treatment with AgNPs significantly decreased the count of viable cells. However, coating AgNPs with albumin significantly improved this viability, in comparison to the results of naked AgNPs, but was still significantly lower than that of the control group. Treatment with AlbNPs did not affect BM cell viability. Similarly, the proportion of apoptotic cells was significantly higher in AgNPs-treated group than that of other groups. Most of these cells were late apoptotic. On the other hand, treatment with AlbNPs affected neither apoptosis nor necrosis of treated BM cells, when compared with the control cells. The genotoxic effect of NPs was tested in BM cells by measurement of DNA damage by comet assay. Fig. 3D shows representative images from different groups. These images were quantitatively analyzed in Fig. 3E. The control group displayed BM cells with intact tail and a full head size. Cells of the AlbNPs group looked like that of the control group but with very short tail migration. Cells of the group treated with AgNPs displayed a short head and a lengthy tail migration. The Alb/AgNPs-treated group had a fully developed head and a slightly moved tail. The tail length, tail DNA, tail moment, and olive moment displayed in Fig. 3E were used to record DNA damage. Comparing cells of the AgNPs-treated group to the other investigated groups showed that they had increases in different parameters of comet assay, indicating more DNA damage and more apoptosis.

NPs also had an impact on the expression level of apoptotic and anti-apoptotic genes (Fig. 3F). When comparing AgNP-treated cells to the control group, the results revealed an intriguing upregulation of Bax and p53 gene expression, while the expression of the antiapoptotic Bcl-2 gene showed no change. The expression of these genes was improved by coating AgNPs with albumin. Yet, their expression was significantly higher, compared with that of the control group. Treatment of BM cells with AlbNPs did not affect the expression of apoptotic genes.

In summary, the results revealed that metallic AgNPs caused apoptosis to bone marrow cells, while the viability of AlbNP-treated BM cells was similar to that of the control group.

#### 3.3.2 Cell cycle assay

**Fig. 4:**
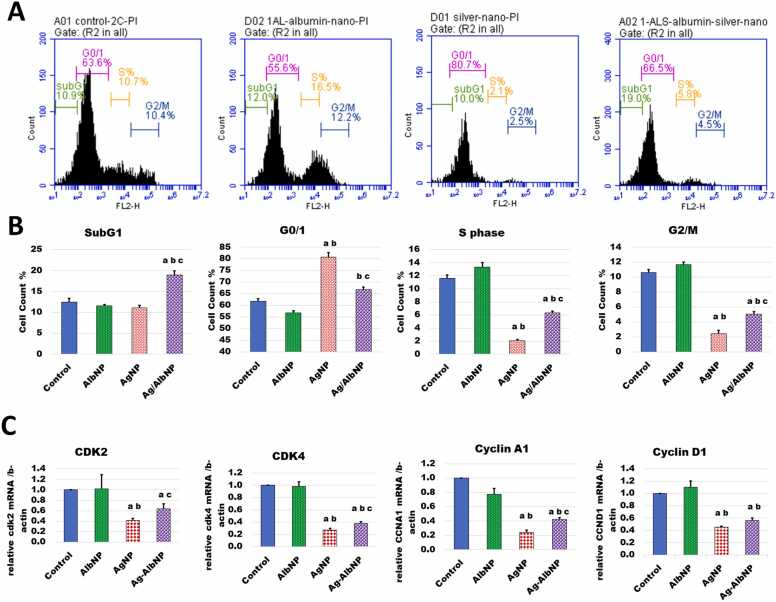
Silver nanoparticles cause cell cycle arrest at G0/1 and downregulate cell cycle genes. Bone marrow was excised from rats ip. administered different NP preparations 3 times a week for 3 weeks, The cell cycle was investigated by flow cytometry and gene expressions analyses. (A) Representative plots of flow cytometric analysis of cell cycle in bone marrow cells treated with different NPs. The DNA content (x axis) is plotted against cell numbers (y axis). The percentage of cells in different cell cycle phases was assessed. M1 = <2 n DNA (Sub G1 cells); M2 = 2 n DNA (G0/G1 cells); M3 = 2–4n DNA (cells in S phase); M4 = 4 n DNA (G2/M cells). (B) Effect of different NPs on bone marrow cell populations in different cell cycle phases. The presented data is a summary of n = 3 flow cytometric analyses. (C) Effect of different NPs on the cell cycle gene expression as determined by quantitative RT-PCR analysis. *Statistical analysis:* Data is presented as mean ± SEM of N = 5. ANOVA p < 0.05. The letters a, b, and c denote a significantly different value from that of control, AlbNPs, and AgNPs-treated groups, respectively (post hoc *t*-test).

Flow cytometric analysis was performed to examine the impact of treatment with various nanoparticles on the progression of the cell cycle of rat bone marrow cells. Fig. 4A is a representative of the resulting plots, and Fig. 4B is a quantitative analysis of different cell cycle phases of n = 3 from each group. In both the control and AlbNPs-treated groups, the population of cells grew from subG1 to G0/G1 phases and then proceeded to divide actively in the subsequent phases. In contrast, the AgNPs- and AgAlb/NPs-treated groups displayed a cell cycle arrest in the G0/1 phase of the cell cycle followed by a decrease in cell population in subsequent S and G2/M phases. These differences were statistically significant. Coating of AgNPs with albumin significantly increased cells in S and G2/M phases, when compared with the results of naked AgNPs. The results revealed also similarities of cell cycle outlook between the control BM cells and the AlbNPs-treated cells.

To understand the events of this BM cell cycle arrest by AgNPs, quantitative RT-PCR was exploited to analyze the expression of some important cell cycle genes in BM cells of different NP-treatments (Fig. 4C). The results revealed that the expression of *Cdk2, Cdk4*, and the cyclins A1 (*Ccna1*) and D1 (*Ccnd1*) genes were significantly downregulated by AgNP-treatment. Coating with albumin did not alter this down regulation but improved it in comparison to naked AgNPs. However, treatment with albumin nanoparticles did not change the expression of these genes in comparison with that of the untreated control.

#### 3.3.3 Effect of different NPs treatments on the differential count of rat bone marrow cells

A differential count test was carried out to determine the impact of the prepared different NPs on the bone marrow cell types. Bone marrow smears were prepared, stained with Giemsa stain, and then viewed under a light microscope to determine the differential count of the cells (Table 2). The data indicates a decrease in most cell types in AgNP-treated group, compared to the results of control and AlbNP-treated group. This difference was significant for RBCs, myeloblasts, megakaryocytes, mast cells, acidophils, and monocytes count. Coating AgNPs with albumin caused a non-significant improvement in differential cell counts.

**Table 2:** Effect of different nanoparticles on the differential count of rat bone marrow cells.

	Control	AlbNPs	AgNPs	Ag/AlbNPs
RBCs (x10^6^/mm^3^)	7.6 ± 0.5	6.2 ± 0.7	4.4 ± 0.5 * #	5.8 ± 0.6 *
Myeloblasts (x10^3^/mm^3^)	6.8 ± 0.6	5.6 ± 1.3	2.6 ± 1.0 *	4.6 ± 0.9 *
Megakaryocytes (x10^3^/mm^3^)	1.8 ± 0.5	1.2 ± 0.2	0.4 ± 0.24 * #	0.8 ± 0.2 *
Mast cells (x10^3^/mm^3^)	1.4 ± 0.4	0.2 ± 0.2 *	0.6 ± 0.2 *	0.6 ± 0.2 *
Acidophils (x10^3^/mm^3^)	4.0 ± 1.3	2.6 ± 0.6	0.6 ± 0.4 * #	1.2 ± 0.6 *
Neutrophils (x10^3^/mm^3^)	0.4 ± 0.2	0.2 ± 0.2	0.2 ± 0.3	0.8 ± 0.4
Basophils (x10^3^/mm^3^)	2.4 ± 1.2	4.0 ± 2.1	4.6 ± 1.8	1.8 ± 0.6
Lymphocytes (x10^3^/mm^3^)	9.2 ± 0.6	9.8 ± 1.9	7.2 ± 1.7	8.2 ± 1.5
Monocytes (x10^3^/mm^3^)	2.8 ± 1.4	2.5 ± 1.2 *	1.5 ± 0.75 *	1.6 ± 0.8 *

Data are expressed as the mean ± SEM. The “* ” denotes significantly different value from the control group; “#” denotes significantly different value from the value of the AlbNPs group.

## 4 Discussion

Bone marrow cells are negatively impacted by conventional chemically manufactured NPs, as demonstrated by prior *in vitro* and *in vivo* studies [8]. For this reason, a lot of attention is focused on the green biosynthesis of NPs from biological sources like microorganisms [30], [51]. In this work, the synthesis of AgNPs was carried out using *E. Coli* D8 crude metabolite, which roles were reducing Ag^+^ ions into metallic silver and stabilizing the NP surface [5]. Based also on previous experience [20], [24], AgNPs, even greenly synthesized, were toxic to many systems and pass through the placenta to be toxic to fetus. Here, we assumed that masking these metallic NPs with a biocompatible material, like albumin, may alleviate their toxicity. Thus, Ag/AlbNPs were produced by capping the prepared AgNPs with albumin. We investigated the effect of these nano preparations on bone marrow cells, and compared their effect with the effect of AlbNPs, which are biodegradable and biocompatible non-metallic NPs.

The prepared NPs were characterized, and the results showed similarities with other previous studies [24], [52] that reported that the UV spectrum of AgNPs displayed a band between 412 and 500 nm, confirming the existence of surface plasmon resonance in Ag metal due to Ag^+^ reduction. Albumin acted as a coating shield to stabilize the surface of AgNPs. AgNPs and AlbNPs were identified by a prominent peak at 249 and 255 nm, respectively, while Ag/AlbNPs UV spectrum displayed a band between 400 and 800 nm. These results agree with that of previous studies [53], [54]. We were able to confirm that albumin is affiliated with AgNPs by comparing the FTIR spectra of AgNPs and AlbNPs with Ag/AlbNPs. We found that the FT-IR spectrum of functional groups detected in the Ag/AlbNPs sample was identical to that of the AgNPs sample as well. The typical characteristic IR peaks of AlbNPs were observed at 3317, 2851, 1635, and 1107 cm-1 attributed to the amine, amide, and amino groups [53], [55]. Surface charge was estimated using zeta potential measurements of the produced nanoparticles. According to our observations, the prepared NPs have a negatively charged zeta potential. The Ag/AlbNPs surface's negatively charged zeta potential results from the accumulation of negatively charged proteins that interact with AgNPs through electrostatic interactions [56], [57]. A negatively charged zeta potential implies stability of NPs. Negatively charged NPs repel each other, which helps to keep them dispersed and stable in suspension, preventing aggregation [56]. The negative charge can also influence the interaction of NPs with biological systems, potentially affecting their uptake and distribution within the body.

Previous studies verified AgNPs' cytotoxicity [58] and genotoxicity [59] through reactive oxygen species (ROS)-mediated apoptosis and necrosis mechanisms. It was reported that the antioxidant defense system activity is halted by excessive levels of ROS brought on by AgNPs’ accumulation inside the cell [60]. The oxidative stress originated by ROS leads to damage to biomolecules including DNA, protein, and lipid [61]. The DNA damage prevented DNA replication and thereby induced cell cycle arrest and apoptosis [62]. Albumin has been shown in earlier research to reduce AgNPs cytotoxicity [63]. Given that albumin is a blood protein that is biocompatible, stable, and non-toxic [64], [65] it also offers an excellent protein capping for AgNPs [57], [66]. In agreement with previous reports [67], [68], we have shown that AgNPs causes cell cycle arrest at the G0/G1 phase, indicating that the cell entered the checkpoint for DNA damage repair and failed to pass the checkpoint exposed to apoptosis, as evidenced by the high cell population of 80.7 % in this phase, which is followed by a decrease in cell population in the following phases [69]. Comparing our results with those of other groups, we found that the Ag/AlbNPs and AlbNPs-treated groups had cell populations in the GO/G1phase of 66.5 % and 55.6 %, respectively, supporting the theory that protein capping reduces the toxicity of silver. Treatment with AgNPs also modified the transcriptional expression linked to the progression of the cell cycle. AgNPs suppressed cyclin D1 expression, in agreement with previous studies [70], [71]. This prevents the phosphorylation of CDK4, followed by the inactivation of the cyclinD-CDK4 complex, thereby inhibiting the progression of the cell cycle. Additionally, AgNPs suppressed cyclin A1 *Ccna1*, and the gene expression of its catalytic CDK2 subunit (*Cdk2*). This prevents the formation of the cyclinA1-CDK2 complex and the phosphorylation series of other cyclins accountable for DNA replication [72].

The present results revealed that AgNPs had the ability to cause bone marrow cells to undergo apoptosis. In contrast to other groups, cells incubated with AgNPs displayed a decrease in viability, an increase in late apoptosis, and a higher percentage of necrosis [62], [73]. Previous studies [74], [75] suggested that AgNPs can induce apoptosis through several mechanisms, including the intrinsic route, which is dependent on anti-apoptotic (Bcl2) and proapoptotic (P53, Bax) proteins. AgNPs interact with membrane proteins and disrupt mitochondrial membrane permeability. This resulted in an increase in reactive oxygen species (ROS) and oxidative stress, which triggered the JNK signaling pathway. This pathway is responsible for upregulating *p53* [76] and *Bax* [77], and downregulating *Bcl2* [78]. The preceding processes resulted in the release of cytochrome c into the cytoplasm, which in turn triggered the caspases cascade and ultimately led to apoptosis [79]. These findings are consistent with our findings, as the AgNPs treated group displayed up-regulated expression of *p53* and *Bax* compared to other studied groups, whereas the expression of the anti-apoptotic *Bcl2* was not affected.

The comet assay was employed in previous works to identify DNA damage caused by ROS generated by AgNPs [80]. In comparison to other groups, our results revealed that the AgNPs-treated group had a higher percentage of DNA damage in the tail DNA, tail length, tail moment, and olive moment, indicating that hematopoietic system is extremely impacted by AgNPs cytotoxicity. According to our research, AgNPs depleted the count of RBCs, myeloplasts (the granulocytic series precursors), megakaryocytes (the platelets precursors), and lymphocytes. These findings agree with previous studies [48]. This result can be attributed to the AgNPs' ability to interact with the cell membranes of the previously mentioned cells, causing membrane damage, releasing ROS, and disrupting the mitochondrial membrane permeability, all of which led to cell death [81]. Numerous studies have demonstrated that AgNPs exhibit greater toxicity, interaction, and penetration into the cell membrane at smaller sizes compared to larger particles [51], [82]. This provided support for our research, since the greenly produced AgNPs size determined by TEM ranged from 8 to 40 nm, allowing for easier and more frequent cell penetration than the Ag/AlbNPs group, which had size ranges of 40–220 nm. Therefore, the AgNPs treated group exhibits a high intracellular concentration of Ag, in contrast to the Ag/AlbNPs treated group. Endocytosis is one of the ways that AgNPs enter the cell [83]. Ultrastructural analysis showed that AgNPs were taken up by lysosomes as soon as they entered the cell, allowing the other free particles to penetrate the nuclear pores, clump the heterochromatin, and damage DNA [84]. Aside from dilation of the endoplasmic reticulum cisternae, certain NPs could infiltrate the mitochondria and change the usual cristae structure [85].

Conjugation with albumin can impact the biological effects of AgNPs. Albumin coating was shown in the present study to significantly reduce the cytotoxic effects of AgNPs on bone marrow cells. This is consistent with a previous study that Albumin coating reduces the cytotoxic effects of AgNPs on human adipose-tissue-derived mesenchymal stem cells. This is beneficial for reducing potential side effects in biomedical applications [86]. However, other effects should be considered such as albumin coating may alter the antibacterial activity of AgNPs. While albumin coating improves the stability of AgNPs, it can also reduce their antibacterial efficacy since the binding of silver ions to albumin may decrease the availability of silver ions, which are crucial for their antimicrobial action [87]. Thus, the interaction between albumin and AgNPs can alter the nanoparticles' behavior in biological systems. This can affect their distribution, clearance, and overall therapeutic potential [88]. Finding the right balance is crucial. In our present work, this coating was beneficial, since it reduced AgNPs toxicity to bone marrow cells. However, in other utilizations where AgNPs are effective, for example, against bacteria without causing significant cytotoxicity, a therapeutic window might be narrow when albumin is present. Taken together, while albumin conjugation can reduce cytotoxicity, it may also compromise the antibacterial properties of AgNPs. It is a trade-off that needs careful consideration depending on the intended application.

In conclusion, the present study reveals that bone marrow is affected by NPs *in vitro* and *in vivo*. Metallic AgNPs are toxic to bone marrow cells, causing BM cell cycle arrest and apoptosis. Numerous parameters, including shape, size, surface charge, and surface coating, may influence the toxicity of the generated nanoparticles [19]. The present study revealed that AgNPs' surface coating with albumin improved the biocompatibility of the nanoparticles with BM cells and diminished the toxicity of AgNPs, which could be attributed to reducing the amount of atomic Ag released from the surface [89], one of the toxicity mechanisms of AgNPs. AlbNPs, in contrast, revealed far less toxicity due to this biocompatibility. It will be healthier to use biocompatible degradable nanoparticles like albumin NPs than metal NPs like AgNPs.

## CRediT authorship contribution statement

**Hyder Ayman:** Writing – review & editing, Supervision, Methodology, Formal analysis, Conceptualization. **Eldabousy Ehdaa:** Writing – original draft, Data curation. **Habbak Lotfy:** Writing – review & editing, Supervision.

## Declaration of Competing Interest

The authors declare that they have no known competing financial interests or personal relationships that could have appeared to influence the work reported in this paper

## Data Availability

Data will be made available on request.
