# Supplementary figures and images for: Apoptosis and cell cycle arrest of bone marrow cells by green-synthesized silver but not albumin nanoparticles
Source: Toxicol Rep. 2025 Feb 13;14:101960. doi: 10.1016/j.toxrep.2025.101960 (PMC11872133; doi:10.1016/j.toxrep.2025.101960)

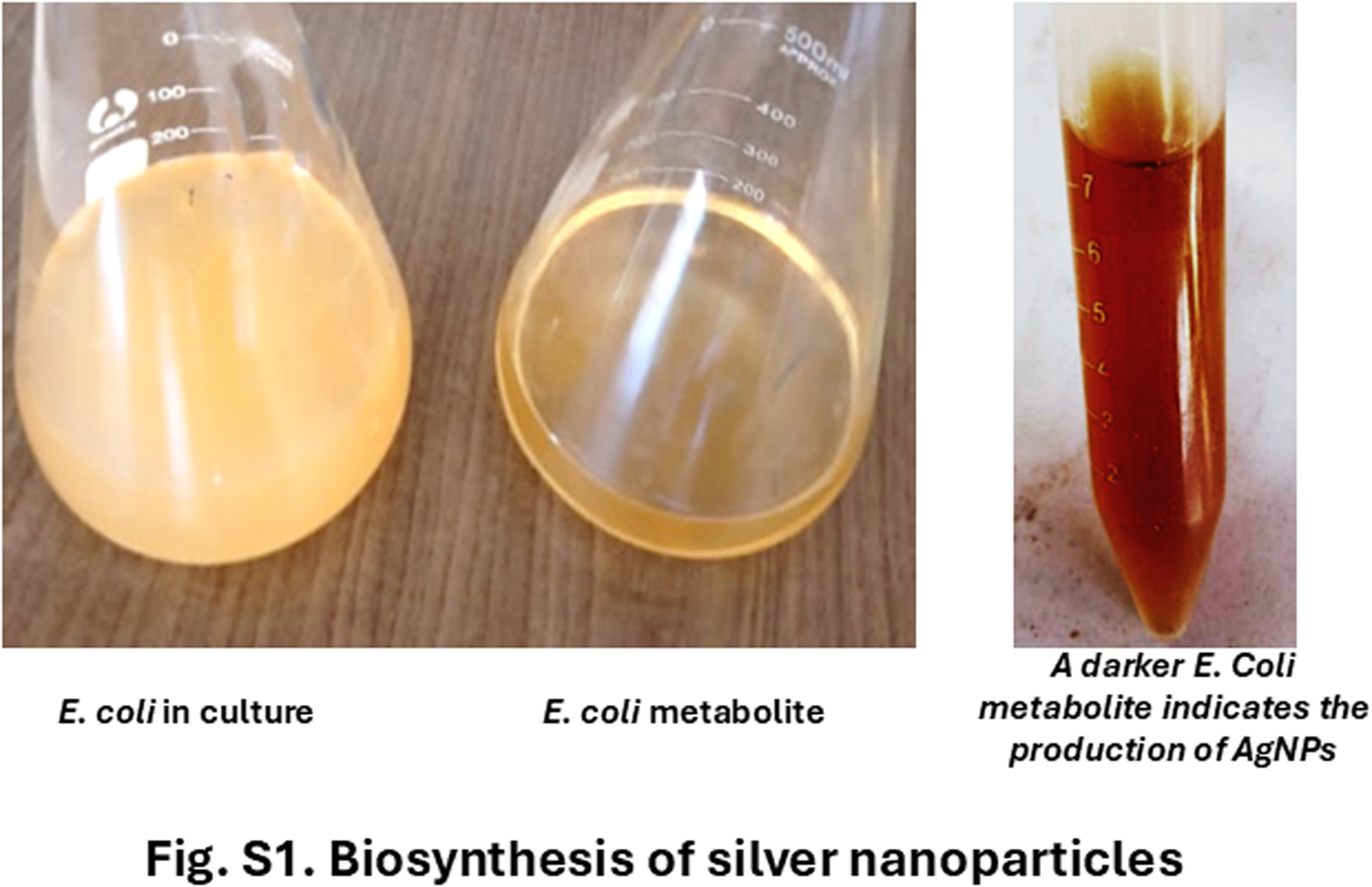

Supplement: Supplementary file 1 — Supplementary material [file mmc1.jpg]
